# A Recombinant Hepatitis C Virus Genotype 1a E1/E2 Envelope Glycoprotein Vaccine Elicits Antibodies That Differentially Neutralize Closely Related 2a Strains through Interactions of the N-Terminal Hypervariable Region 1 of E2 with Scavenger Receptor B1

**DOI:** 10.1128/JVI.00810-19

**Published:** 2019-10-29

**Authors:** Janelle Johnson, Holly Freedman, Michael Logan, Jason Alexander Ji-Xhin Wong, Darren Hockman, Chao Chen, Jianqi He, Michael R. Beard, Nicholas S. Eyre, Thomas F. Baumert, D. Lorne Tyrrell, John L. M. Law, Michael Houghton

**Affiliations:** a Li Ka Shing Institute of Virology, Department of Medical Microbiology and Immunology, University of Alberta, Edmonton, Alberta, Canada; b Department of Molecular and Biomedical Science, Research Centre for Infectious Diseases, University of Adelaide, Adelaide, SA, Australia; c Inserm U1110, Institut de Recherche sur les Maladies Virales et Hépatiques, Université de Strasbourg, Strasbourg, France; d Pôle Hepato-Digestif, Institut Hopitalo-Universitaire, Hôpitaux Universitaires de Strasbourg, Strasbourg, France; University of Southern California

**Keywords:** hypervariable region 1, scavenger receptor B1, genotype 2a, hepatitis C virus, isolate-specific, neutralizing antibodies, vaccine

## Abstract

The global health burden for hepatitis C virus (HCV) remains high, despite available effective treatments. To eliminate HCV, a prophylactic vaccine is needed. One major challenge in the development of a vaccine is the genetic diversity of the virus, with 7 major genotypes and many subtypes. A global vaccine must be effective against all HCV genotypes. Our previous data showed that the 1a E1/E2 glycoprotein vaccine component elicits broad cross-neutralizing antibodies in humans and animals. However, some variation is seen in the effectiveness of these antibodies to neutralize different HCV genotypes and isolates. Of interest was the differences in neutralizing activity against two closely related isolates of HCV genotype 2a, the J6 and JFH-1 strains. Using site-directed mutagenesis to generate chimeric viruses between the J6 and JFH-1 strains, we found that variant amino acids within the core E2 glycoprotein domain of these two HCV genotype 2a viruses do not influence isolate-specific neutralization. Further analysis revealed that the N-terminal hypervariable region 1 (HVR1) of the E2 protein determines the sensitivity of isolate-specific neutralization, and the HVR1 of the resistant J6 strain binds scavenger receptor class-B type-1 (SR-B1), while the sensitive JFH-1 strain does not. Our data provide new information on mechanisms of isolate-specific neutralization to facilitate the optimization of a much-needed HCV vaccine.

**IMPORTANCE** A vaccine is still urgently needed to overcome the hepatitis C virus (HCV) epidemic. It is estimated that 1.75 million new HCV infections occur each year, many of which will go undiagnosed and untreated. Untreated HCV can lead to continued spread of the disease, progressive liver fibrosis, cirrhosis, and eventually, end-stage liver disease and/or hepatocellular carcinoma (HCC). Previously, our 1a E1/E2 glycoprotein vaccine was shown to elicit broadly cross-neutralizing antibodies; however, there remains variation in the effectiveness of these antibodies against different HCV genotypes. In this study, we investigated determinants of differential neutralization sensitivity between two highly related genotype 2a isolates, J6 and JFH-1. Our data indicate that the HVR1 region determines neutralization sensitivity to vaccine antisera through modulation of sensitivity to antibodies and interactions with SR-B1. Our results provide additional insight into optimizing a broadly neutralizing HCV vaccine.

## Abstract (precis)

A vaccine is still urgently needed to overcome the hepatitis C virus (HCV) epidemic. It is estimated that 1.75 million new HCV infections occur each year, many of which will go undiagnosed and untreated. Untreated HCV can lead to continued spread of the disease, progressive liver fibrosis, cirrhosis, and eventually, end-stage liver disease and/or hepatocellular carcinoma (HCC). Previously, our 1a E1/E2 glycoprotein vaccine was shown to elicit broadly cross-neutralizing antibodies; however, there remains variation in the effectiveness of these antibodies against different HCV genotypes. In this study, we investigated determinants of differential neutralization sensitivity between two highly related genotype 2a isolates, J6 and JFH-1. Our data indicate that the HVR1 region determines neutralization sensitivity to vaccine antisera through modulation of sensitivity to antibodies and interactions with SR-B1. Our results provide additional insight into optimizing a broadly neutralizing HCV vaccine.

## INTRODUCTION

There are about 71 million individuals chronically infected with hepatitis C virus (HCV) worldwide, and about 1.75 million new infections are estimated to occur each year (1, 2). HCV remains a global health burden today despite newly available therapeutic drugs. Untreated HCV infection can lead to progressive liver fibrosis and cirrhosis and in some cases, eventually, end-stage liver disease or hepatocellular carcinoma (HCC), resulting in about 400,000 deaths annually (1, 3). It is estimated that 25% of all HCC and its health complications arising from advanced cirrhosis are the result of HCV infection (3–5).

HCV is a highly diverse virus with seven major genotypes and 67 characterized subtypes (5–8). HCV displays greater sequence diversity than even human immunodeficiency virus (HIV); genotypes can differ by up to 30% in nucleotide sequence, and subtypes by up to 15% (5, 9). Even HCV isolates within the same subtype can have sequence variance of up to 10%. This large sequence variation in HCV has been an ongoing challenge in the development of vaccines. While direct-acting antivirals (DAAs) are capable of curing HCV in over 90% of cases, there remain challenges in the treatment of HCV, such as the cost and access to these drugs, as well as the lack of protective immune responses in cured patients leading to the risk of reinfection (4, 10). Additionally, some patients cured with DAA who have advanced fibrosis and cirrhosis at the time of treatment also remain at elevated risk for the development of HCC, despite being cured of HCV (11–13). Therefore, a vaccine to prevent HCV is still urgently needed.

The presence of neutralizing antibodies has been shown to correlate with protection from HCV infection *in vivo* (14, 15). Isolation of antibodies capable of inhibiting infection of a broad range of HCV genotypes highlighted the protective role of neutralizing antibodies in the prevention of HCV infection (16). Subsets of these antibodies have been shown to neutralize both homologous and heterologous HCV genotypes by targeting various regions of the envelope 1 (E1) and E2 proteins. Many of these antibodies target conserved regions within the E2 protein that interact with the cluster of differentiation 81 (CD81) HCV receptor (17–19). However, there are neutralizing epitopes comprising both E1 and E2 targeted by two strongly cross-neutralizing antibodies within antigenic region 4A (AR4A) and AR5A (19). Examples of HCV evading the neutralizing antibody response have been reported. Mutations in the E1 and E2 proteins can result in escape from broadly neutralizing monoclonal antibodies (reviewed in reference 16). Some of these mutations also alter virus interactions with entry receptors CD81 and scavenger receptor class B type 1 (SR-B1) (20, 21).

HCV entry is a complex process involving both the viral envelope proteins, lipoproteins present on the virion, and a large number of cell surface proteins and receptors (1, 22). Initial attachment of lipoprotein-associated HCV virions to the cell surface is through interactions with heparan sulfate glycosaminoglycans (GAG) and low-density lipoprotein receptor. Virions subsequently bind with SR-B1 in a stepwise process involving lipoproteins and the HCV E2 protein (22–24). Binding to SR-B1 is thought to induce subsequent binding of the E2 protein to CD81, although the mechanism of this transition is not well understood (22, 25). The interaction with CD81 triggers a signaling cascade that results in recruitment of actin to the cell surface and further trafficking of the virion/receptor complex to the cell-cell tight junctions (1, 22). Within the tight junctions, interactions with claudin-1 (CLDN1) and occludin (OCLN) allow the virion to enter the cell via clathrin-mediated endocytosis (22). HCV E2 protein interactions with the CD81 receptor have been characterized. It has been shown that recombinant E2 binds directly to CD81, and specific E2 amino acid residues involved in CD81 binding have been identified (25–29). On the other hand, the interaction between E2 and the SR-B1 receptor is complex and involves accessory interactions with lipoproteins on the virion as well as direct interaction with the E2 protein thought to be mediated by hypervariable region 1 (HVR1), the 27-amino-acid sequence at the amino (N) terminus of the E2 protein (23, 24, 30, 31). Direct interaction of soluble E2 protein with SR-B1 has been observed for the genotype 1a H77, 1b BK, 2a J6, and 2a JFH-1 virus strains (30–32). Deletion of HVR1 negates soluble E2/SR-B1 binding and results in resistance to antibodies targeting SR-B1 during infection (30, 31, 33, 34).

HVR1 is also an immunodominant decoy with high diversity utilized as an escape mechanism during natural HCV infection (35). HVR1 rapidly evolves under immune pressure *in vivo*, and this rapid evolution results in HVR1-specific antibodies that are strain specific (35, 36). Despite HVR1 having such high sequence diversity, there is evidence of amino acid charge conservation, indicating a conserved function of HVR1 (37). Recently, HVR1 has been shown to be important in genotype differences in neutralization sensitivity to monoclonal antibodies (31, 33, 38, 39). Deletion of HVR1 drastically increases the neutralization sensitivity of a broad range of HCV genotypes as well as decreasing genotype-specific neutralization sensitivity to monoclonal antibodies.

An E1/E2 glycoprotein vaccine has been tested in chimpanzees, and the elicited immune response was able to prevent infection by homologous virus challenge (40). Additionally, while this vaccine was unable to prevent acute infection of a heterologous strain, there was a significant reduction in the rate of chronic infections postvaccination, and elicited antibodies were capable of neutralizing a diverse range of HCV genotypes *in vitro* (41, 42). No other HCV vaccine candidate has been shown to be potent in the chimpanzee model. Clinical testing of this first-generation recombinant 1a E1/E2 glycoprotein vaccine demonstrated safety in humans (43) and showed the capacity to elicit strong lymphoproliferative response and antibody responses capable of neutralizing representatives of all genotypes of HCV *in vitro* (44). The antibody response elicited by this 1a E1/E2 vaccine candidate was further characterized by studies of antisera from vaccinated animals (45, 46). Previously, we found that sera from vaccinated animals has an antibody response similar to that observed in humans. In addition, we determined that these antibodies do compete with broadly neutralizing monoclonal antibodies for binding epitopes throughout the E1 and E2 proteins. However, there is variation in the effectiveness of these vaccine-induced antibodies to neutralize HCV across genotypes with strong neutralization against viruses of genotypes 1, 4, 5, and 6 but reduced effectiveness against genotypes 2, 3, and 7.

In the present study, we show that while the recombinant 1a E1/E2 vaccine-induced antisera from a vaccinated goat (45) exhibit low neutralization against the genotype 2a J6 virus, the antisera can efficiently neutralize JFH-1, a closely related genotype 2a virus. We found that the E2 glycoprotein largely mediates this observed differential neutralization, with the E2 HVR1 being a major determinant in the isolate-specific neutralization sensitivity between these two genotype 2a isolates. Surprisingly, HVR1 appears to be acting indirectly to mediate this differential neutralization and not via HVR1-specfic neutralizing antibodies. J6 HVR1 but not JFH-1 HVR1 is shown to bind directly to SR-B1, which might contribute to its observed neutralization resistance.

## RESULTS

### A major determinant in neutralization sensitivity between J6 and JFH-1 isolates is located in the E2 protein.

**FIG 1:**
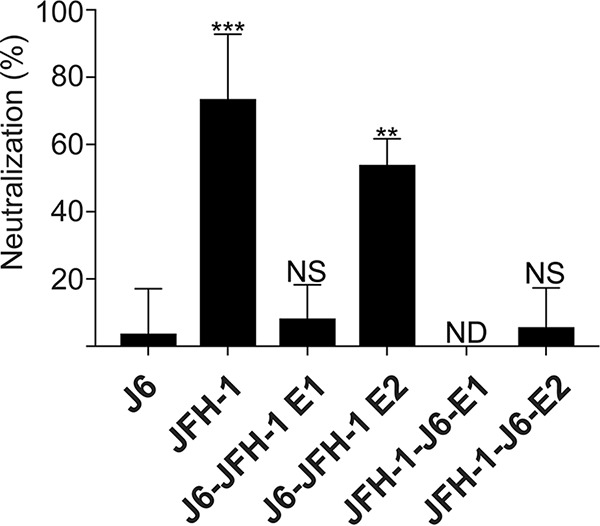
E2 determines the neutralization sensitivity of recombinant HCVcc. The ability of vaccine-induced antisera (1a E1/E2) to neutralize J6, JFH-1, and recombinant forms of the J6 and JFH-1 viruses was tested. All HCVcc stocks were diluted to a titer of 500 TCID_50_/ml and preincubated with heat-inactivated antisera at a dilution of 1/100, and the mixture was then added to Huh7.5 cells. Viruses encode an NS5A-Nano luciferase fusion protein (64). Infection was quantified by measuring the luminescence signal 48 hours after infection. The error bars represent the standard deviation of data from three independent experiments, each performed with triplicate wells. Statistical significance was calculated using a one-way ANOVA followed by a Dunnett’s multiple-comparison test to compare the means of each column to J6 using GraphPad Prism 7 software, and significant differences are indicated above the bars. ***, *P* < 0.001; **, *P* < 0.01; NS, no significance; ND, no data were obtained due to nonviable virus.

Since the E1 and E2 proteins are the known targets for neutralizing antibodies, we investigated if the main determinants of neutralization between J6 and JFH-1 were located within the E1 and/or E2 proteins. The E1 and E2 proteins were exchanged between the relatively resistant J6 virus and the relatively sensitive JFH-1 virus. These recombinant cell culture-derived HCV (HCVcc) viruses were then tested for their neutralization sensitivity to the recombinant 1a E1/E2 vaccine antisera, and their sensitivities were compared (Fig. 1). Similar to previous data from our lab (45), the J6 virus showed a resistant phenotype in which only about 10% of the virus infectivity was neutralized, while the JFH-1 virus showed a more sensitive phenotype with about 64% of the virus infectivity neutralized. While insertion of E1 from JFH-1 into the J6 virus (J6-JFH-1 E1) did not show a significant increase in neutralization sensitivity over the resistant J6 virus, insertion of the E2 from JFH-1 (J6-JFH-1 E2) did show a significant increase in neutralization sensitivity compared to the J6 virus (Fig. 1). Conversely, replacement of the JFH-1 E2 with the E2 protein from J6 (JFH-1-J6 E2) reduced neutralization sensitivity to the level of the J6 virus (Fig. 1). We were not able to test the effect of the E1 of J6 in JFH-1 virus since replacement of the JFH-1 E1 with the E1 protein from the J6 (JFH-1-J6 E1) construct was not viable in cell culture. The E2 protein from JFH-1 was able to confer sensitivity to the J6 virus, while the E2 protein from the J6 virus was able to confer resistance to the JFH-1 virus. These results indicate that the E2 protein is a major determinant of the isolate-specific neutralization sensitivity between the J6 and JFH-1 HCV 2a isolates.

### Variant amino acids throughout E2 are not responsible for the differential neutralization between J6 and JFH-1.

Previous research has demonstrated that polymorphisms of the E2 protein can affect HCV sensitivity to broadly neutralizing antibodies (21, 32, 47–50). Therefore, it was possible that E2 amino acid differences between J6 and JFH-1 HCV 2a isolates could be responsible for the observed difference in neutralization sensitivity.

**FIG 2:**
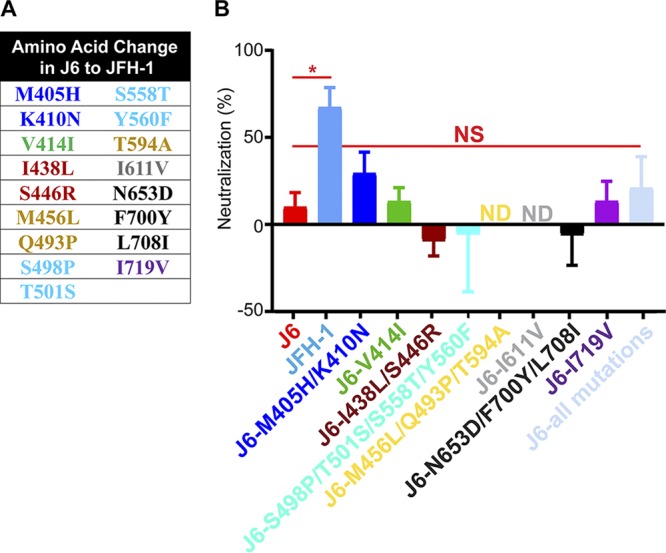
Variant amino acids within the E2 protein do not confer differential neutralization sensitivity between J6 and JFH-1 viruses. (A) Amino acid positions within the J6 background that were exchanged for the JFH-1 amino acid variant. Amino acid positions that were identical between H77 and JFH-1 but differed in J6 were identified, along with three additional amino acid positions of interest, two from the HVR1 region and one from domain 1. Colors represent the groupings of variants according to their spatial location in the E2 protein (28, 29). (B) Variant amino acid residues from the sensitive JFH-1 WT virus were engineered into the resistant J6 virus using site-directed mutagenesis. A total of 500 TCID_50_/ml HCVcc were preincubated with either heat-inactivated pre- or post-1a E1/E2 antisera at a dilution of 1/100, followed by addition to Huh7.5 cells. The antisera/virus inoculum was replaced with fresh medium after 6 hours, and the infection was measured 48 hours postinfection. The error bars represent the standard deviation of data from three independent experiments, each performed within triplicate wells. One-way ANOVA followed by a Dunnett’s multiple-comparison test was calculated comparing the means of each column to the J6 using GraphPad Prism 7 software. *, *P* < 0.05; NS, no significance; ND, no data were obtained due to nonviable virus.

Variant amino acids between J6 and JFH-1 were selected by a comparison of the E2 amino acid sequences. There are 49 variant amino acids between J6 and JFH-1. We then compared the sequence of the J6 and JFH-1 isolates to that of the known sensitive 1a H77 strain that is homologous to the vaccine antigen (Fig. 2A) (45). Amino acids that were identical between the sensitive H77 and JFH-1 strains but variant in the resistant J6 strain were selected for analysis. Furthermore, three additional amino acid positions (405, 410, and 446) were selected based on their potential role in affecting E2 conformation as predicted by computational modeling (28, 29); these are located in the HVR1 region and domain 1 (the 32 amino acids directly downstream of HVR1 as described by Douam et al. [51]). Using this method, 17 variant amino acid residues were identified for further study (listed in Fig. 2A). Groups of the variant amino acid which were close in proximity within the three-dimensional (3D) E2 protein structure from the JFH-1 virus were introduced together into the J6 virus. In addition, a construct was also created where all 17 variant amino acids from the JFH-1 virus were introduced together into the J6 virus. All constructs were tested for their neutralization sensitivity to the 1a E1/E2 vaccine antisera. None of the groups of mutations, including the recombinant virus with all 17 variant residues (pale blue), showed any significant change in neutralization sensitivity from that of the J6 virus (Fig. 2B). Two of the mutation groups (J6-M456L/Q493P/T594A and J6-I611V) were not viable in cell culture (yellow and gray). Interestingly, J6-M405H/K410N virus (dark blue) showed the greatest increase in neutralization sensitivity, but the enhancement was not significant. Together, these data indicate that the variant amino acids that are conserved among the sensitive strains H77 and JFH-1 but differ in the resistant J6 strain are not the major contributor to the difference in neutralization sensitivity to 1a E1/E2 antisera.

### HVR1 is a major determinant of differential neutralization sensitivity between J6 and JFH-1.

**FIG 3:**
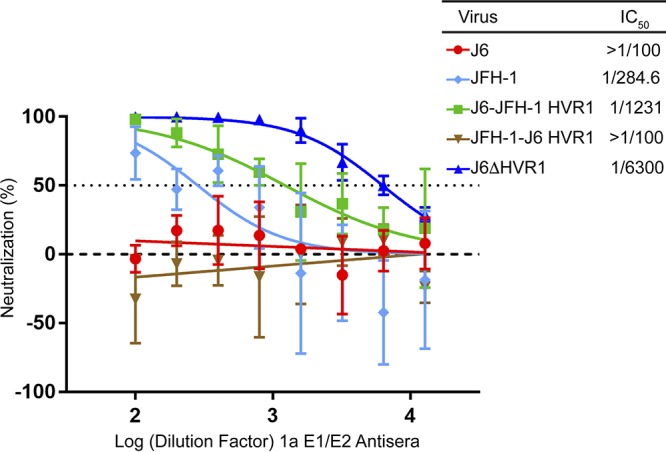
HVR1 is a determinant of isolate-specific neutralization sensitivity. Neutralization sensitivity to 1a E1/E2 vaccine-induced antisera of J6 (red), JFH-1 WT (light blue), J6-JFH-1 HVR1 (green), JFH-J6 HVR1 (brown), and J6-ΔHVR1 (dark blue) viruses. A total of 500 TCID_50_/ml HCVcc along with 2-fold-diluted antisera (between 1/100 and 1/12,800) was preincubated for 1 hour, and mixtures were added to Huh 7.5 cells. The infection was quantified by measuring the luminescence signal at 48 hours postinfection. The error bars represent the standard deviation of data from three independent experiments, each performed with triplicate wells. The IC_50_ was calculated using GraphPad Prism 7 software by finding the nonlinear regression of a variable slope. Statistical differences of JFH-1 WT, ΔHVR1, and HVR1 recombinant viruses from J6 were calculated with GraphPad Prism 7 software using a two-way ANOVA followed by a Dunnett’s multiple-comparison test.

Previous studies have shown that deleting HVR1 (amino acids 384 to 410) results in an increased sensitivity to neutralization by monoclonal antibodies as well as a decrease in the genotype variation to neutralization (31, 33, 38). We therefore investigated the role of the HVR1 in the isolate-specific neutralization sensitivity of the J6 and JFH-1 viruses. HVR1 chimeric viruses were created by swapping the HVR1 domain between the J6 and JFH-1 viruses. We also tested the J6 virus lacking the HVR1 domain; however, the JFH-1 virus lacking HVR1 was not viable in cell culture. The chimeric viruses were then tested for their neutralization sensitivity to 1a E1/E2 antisera. Replacing HVR1 in J6 with HVR1 of JFH-1 (J6-JFH-1 HVR1) resulted in a significant increase in sensitivity to the 1a E1/E2 antisera compared to J6 virus (Fig. 3, green compared to red) and had a sensitivity phenotype more similar to that of the JFH-1 wild-type (WT) virus (light blue). Conversely, replacement of JFH-1 HVR1 with the J6 HVR1 within the JFH-1 virus (JFH-1-J6 HVR1) displays a resistant phenotype similar to that of the J6 virus (Fig. 3, brown compared to red). Deletion of the HVR1 from the resistant J6 virus resulted in a hypersensitive phenotype to our 1a E1/E2 antisera compared to J6 virus (Fig. 3, dark blue). Replacement with JFH-1 HVR1 in J6 recombinant virus shows increased sensitivity compared to J6 virus, indicating that JFH-1 HVR1 confers neutralization sensitivity. Removal of HVR1 (J6-ΔHVR1) further enhances the neutralization sensitivity by 5-fold by a comparison of half-maximal inhibitory concentration (IC_50_) values for J6-JFH-1 HVR1 and J6-ΔHVR1 (1/1,231 for J6-JFH-1 HVR1 compared to 1/6,300 for J6-ΔHVR1). Together, these data indicate that HVR1 is a major determinant of isolate-specific neutralization sensitivity.

### HVR1 of heterologous genotypes is not a direct target of polyclonal antibodies elicited by the 1a E1/E2 vaccine.

**FIG 4:**
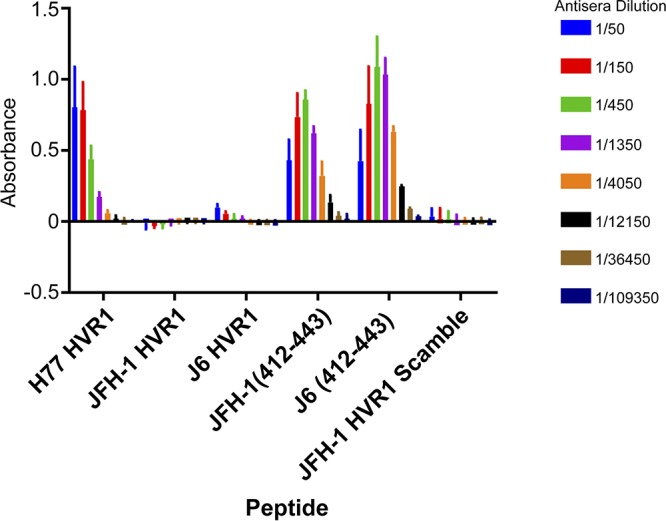
1a E1/E2 antisera do not bind to peptide-encoding HVR1 derived from J6 or JFH-1. The binding of 1a E1/E2 antisera to peptides encoding the HVR1 of 1a H77, 2a JFH-1, and 2a J6 viruses, as well as peptide corresponding to amino acids (aa) 412 to 443 of either JFH-1 or J6 was assessed. Biotinylated peptides were bound to wells coated with neutravidin, and serial-diluted pre- and postvaccinated antisera from 1a E1/E2-vaccinated goat was added. Bound IgG was detected with an anti-goat HRP-conjugated antibody. Absorbance was read at 450 nm. Background binding of presera and postsera to no-peptide control wells was subtracted, and absorbance values were plotted with GraphPad Prism 7 software. Error bars represent the standard deviation of data from three independent experiments, each performed with duplicate wells.

Antibodies targeting HVR1 are capable of neutralizing HCV infection (35, 52). It is possible that antibodies within the 1a E1/E2 antisera specifically target the HVR1 of JFH-1 but not J6, which could account for the observed differential neutralization sensitivity. We therefore wanted to test if antibodies in the antisera were able to recognize the HVR1 sequence of JFH-1 but not J6. The antisera do not bind peptide derived from HVR1 of either J6 or JFH-1 but did show reactivity to HVR1 of H77 (Fig. 4). However, 1a E1/E2 vaccine antisera were reactive to peptide derived from a more conserved region, the 31 amino acids downstream of HVR1 (residues 412 to 443) of both the J6 and JFH-1. This region is known to contain many neutralizing epitopes for broadly neutralizing monoclonal antibodies (16), and our vaccine antisera are known to contain antibodies that target these epitopes (45, 46). These data indicate that vaccine-induced antibodies targeting HVR1 are not responsible for the differential neutralization of J6 and JFH-1 strains but, rather, that HVR1 is playing an indirect role.

### HVR1 influences E1/E2 antibody neutralization.

**FIG 5:**
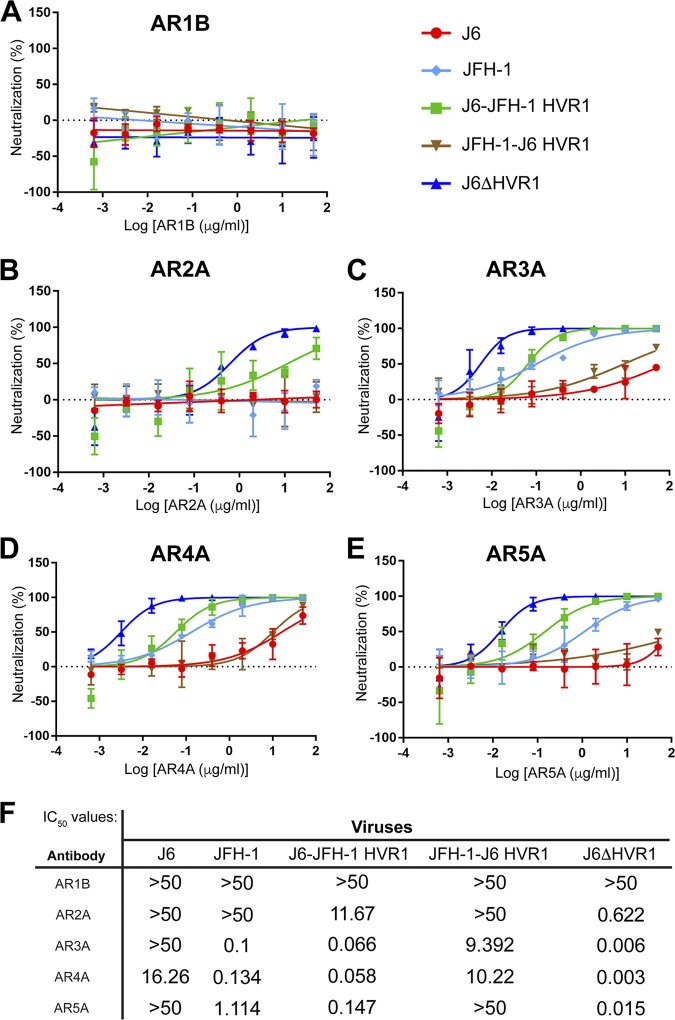
HVR1 modulates sensitivity to monoclonal neutralizing antibodies in an isolate-specific manner. Broadly neutralizing monoclonal antibodies (5-fold dilutions starting with 50 μg/ml) AR1B (A), AR2A (B), AR3A (C), AR4A (D), and AR5A (E) were preincubated with 500 TCID_50_/ml wild-type or modified HVR1 HCVcc for 1 hour, and then the mixture was used to infect Huh7.5 cells for 6 hours. The infection was quantified by measuring the luminescence signal at 48 hours postinfection. The error bars represent the standard deviation of data from three independent experiments, each performed with duplicate wells. (F) IC_50_ values were calculated using GraphPad Prism 7 software by finding the nonlinear regression of a variable slope. Statistical comparisons of all chimeric HVR1 virus to J6 for all antibodies was calculated with a two-way ANOVA followed by a Dunnett’s multiple-comparison test using GraphPad Prism 7 software.

To investigate how HVR1 affects neutralization indirectly, we tested the neutralization sensitivity of HVR1 chimeric viruses using a panel of well-characterized monoclonal antibodies. The antibodies selected bind to a diverse range of epitopes throughout the E2 protein (AR1B, AR2A, and AR3A), as well as conformational epitopes requiring amino acid residues within both E1 and E2 (AR4A and AR5A) (19, 53). It is possible that HVR1 is influencing conformation of the glycoproteins and, subsequently, the exposure of neutralizing epitopes. For AR1B, no neutralization was observed for any of the viruses tested, since this antibody is known to be nonneutralizing against HCVcc (19, 53) (Fig. 5A). For AR2A, the J6-ΔHVR1 virus was sensitive to neutralization, and the J6-JFH-1 HVR1 virus showed moderate sensitivity (Fig. 5B), indicating that AR2A, despite being a 1a isolate-specific antibody, can recognize J6 virus when it contains JFH-1 HVR1 or has no HVR1. For AR3A, AR4A, and AR5A, a shared pattern of neutralization sensitivity was observed (Fig. 5C to E). J6-ΔHVR1 virus was hypersensitive to all three antibodies. J6-JFH-1 HVR1 virus showed a sensitive phenotype similar to that of the JFH-1 WT virus, and JFH-J6 HVR1 virus showed a relative resistant phenotype similar to that of the J6 virus. IC_50_ values for neutralization of HVR1 chimeric viruses by monoclonal antibodies are shown in Fig. 5F. These data mirror the sensitivity of chimeric viruses to 1a E1/E2 antisera observed in Fig. 3. A general trend in sensitivity is observed using monoclonal antibodies as well as 1a E1/E2 vaccine antisera such that virus containing the HVR1 of J6 shows relative resistance, while virus with HVR1 from JFH-1 shows relative sensitivity. This is consistent with the idea that the presence of HVR1 somehow inhibits exposure of a variety of neutralizing epitopes, and in the case of J6-ΔHVR1, removal of this domain exposes these epitopes (38).

### Influence of HVR1 on viral interactions with entry receptors CD81 and SR-B1.

**FIG 6:**
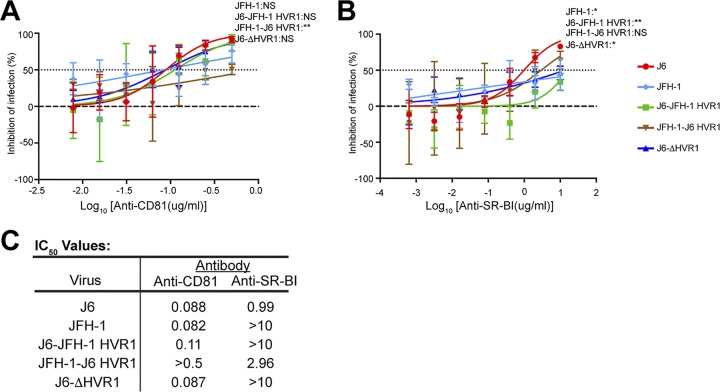
WT and HVR1-modified J6 and JFH-1 virus response to inhibition by anti-CD81 and anti-SR-B1. Anti-CD81 (2-fold serial dilutions starting with a concentration of 0.5 μg/ml) (A) and anti-SR-B1 (5-fold serial dilutions starting with a concentration of 10 μg/ml) (B) were preincubated with Huh7.5 cells for 4 hours. Then 500 TCID_50_/ml HCVcc was added to Huh7.5 cells for 6 hours, followed by replacement with fresh medium. Infection quantified by measuring luminescence signal was detected 48 hours postinfection. Error bars represent the standard deviation of data from three independent experiments, each performed within duplicate wells. Statistical significance of the different viruses’ inhibition sensitivity for each dilution was calculated with a two-way ANOVA using GraphPad Prism 7 software, and *P* values for differences from the J6 virus are displayed for the highest dilution of both anti-CD81 (0.5 μg/ml) and anti-SR-B1 (10 μg/ml).**, *P* < 0.01; *, *P* < 0.05; NS, no significance. (C) IC_50_ values were calculated from dilution curves using GraphPad Prism 7 software to find the nonlinear regression of a variable slope.

We hypothesized that HVR1 might also affect virus-receptor interactions. Therefore, we investigated if HVR1 differentially affects the CD81-dependent or SR-B1-dependent entry of J6 and JFH-1 virus. The inhibition of virus entry by anti-CD81 was not significantly different between J6 and JFH-1 or between J6 and either J6-JFH-1 HVR1 or J6-ΔHVR1 (Fig. 6A). However, JFH-1 virus containing the HVR1 of J6 (JFH-1-J6 HVR1) showed reduced sensitivity to anti-CD81 antibody inhibition compared to both J6 and JFH-1 virus (Fig. 6A). IC_50_ values were similar, with the exception of those for JFH-1-J6 HVR1 virus (Fig. 6C). These data indicate a potential strain-specific role for HVR1 in CD81 engagement.

**FIG 7:**
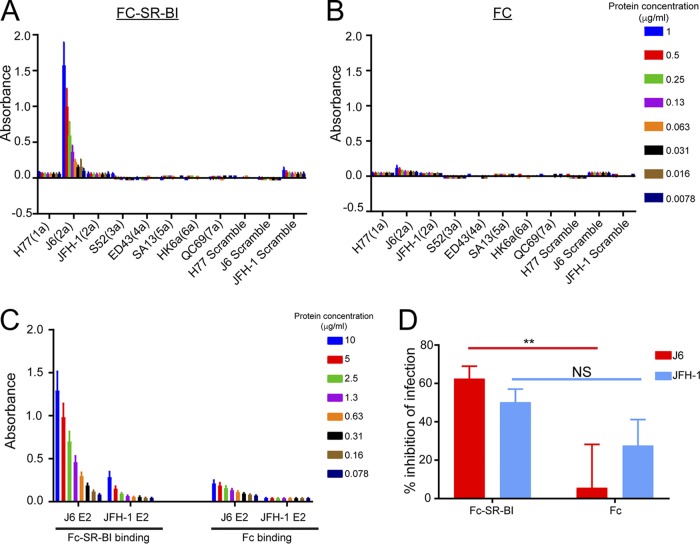
SR-B1 protein specifically binds J6 HVR1. The binding of Fc-tagged SR-B1 (A) and human Fc (B) proteins to peptides encoding the HVR1 of the 1a H77, 2a J6 or 2a JFH-1, 3a S52, 4a ED43, 5a SA13, 6a HK6a, and 7a QC69 HVR1 sequences, in addition to scramble controls of the 1a H77, 2a J6, and 2a JFH-1 HVR1 peptides, was assessed. Biotinylated peptides were bound to wells coated with neutravidin. Two-fold serial-diluted Fc-tagged SR-B1 protein was added to the plate coated with peptides (with a starting concentration of 1 μg/ml). Bound protein was detected with an HRP-conjugated anti-Fc antibody. Error bars represent the standard deviation from three independent experiments, each performed within duplicate wells. Statistical differences from scramble control peptide were calculated with a two-way ANOVA using GraphPad Prism 7 software. (C) The binding of the Fc-SR-B1 and Fc protein to J6- and JFH-1-soluble E2 was assessed. J6- and JFH-1-soluble E2 were coated on plates. Two-fold serial-diluted Fc-tagged SR-B1 protein was added to the plate coated with peptides (with a starting concentration of 10 μg/ml). Bound protein was detected with an HRP-conjugated anti-Fc antibody. Error bars represent the standard deviation from three independent experiments, each performed within duplicate wells. Statistical differences from Fc alone were calculated with a two-way ANOVA using GraphPad Prism 7 software. (D) Fc-SR-B1 protein and Fc protein alone (2-fold serial dilutions with a starting concentration of 50 μg/ml) were preincubated with 500 TCID_50_/ml J6 and JFH-1 HCVcc for 1 hour, and then the mixture was added to Huh7.5 cells for 6 hours, followed by replacement with fresh medium. Infection was quantified by measuring the luminescence signal detected 48 hours postinfection. Error bars represent the standard deviation of data from three independent experiments, each performed within duplicate wells. Statistical significance of the different viruses’ inhibition sensitivity was calculated with a one-way ANOVA using GraphPad Prism 7 software, and *P* values for comparison of the inhibition by Fc-SR-B1 to Fc protein for the J6 and JFH-1 virus are displayed. **, *P* < 0.01; NS, no significance.

We next tested the effects of HVR1 on SR-B1-dependent entry in a similar fashion. J6 virus clearly showed increased sensitivity to neutralization by anti-SR-B1 compared to JFH-1, J6-JFH-1 HVR1, and J6-ΔHVR1 virus (Fig. 6B). IC_50_ values for J6 and JFH-1-J6 HVR1 virus were more similar than those for virus containing the HVR1 from JFH-1 or lacking HVR1 (Fig. 6C). This indicates a clear role for HVR1 in strain-specific SR-B1 interactions. To explore this further, we tested the direct binding of SR-B1 protein to HVR1 derived from J6 and JFH-1. Strikingly, we found that recombinant Fc-SR-B1 binds specifically to HVR1 of J6, but not to the HVR1 sequence of any of the other genotypes or isolates tested, highlighting a strong interaction of the 2a J6 HVR1 with SR-B1 (Fig. 7A). Additionally, we tested the binding of the Fc-SR-B1 to J6- and JFH-1-soluble E2. In agreement with the binding to the HVR1 peptides, we found a specific interaction with the J6-soluble E2 and minimal binding of the JFH-1 soluble E2 with the Fc-SR-B1 protein (Fig. 7C). As a control, Fc protein alone does not show binding to any peptides or soluble E2 proteins tested (Fig. 7B and C). The functionality of the Fc-SR-B1 protein was investigated using its ability to inhibit the infection of J6 and JFH-1 HCVcc compared to the Fc protein alone (Fig. 7D). The J6 HCVcc was significantly inhibited by the Fc-SR-B1 protein compared to the Fc protein alone at a dilution of 50 μg/ml. For the JFH-1 HCVcc, the difference from the Fc protein alone was not statistically significant. Together, these findings indicate that the HVR1 of J6 has a specific direct interaction with SR-B1.

## DISCUSSION

In this study, the molecular determinants of differential neutralization sensitivity to our 1a E1/E2 vaccine polyclonal antisera were investigated for two highly related HCV isolates, the genotype 2a J6 and JFH-1 viruses. Using wild-type and chimeric viruses derived from both strains, we showed that the differential neutralization was mediated through the E2 envelope glycoprotein (Fig. 1). When variant core E2 domain amino acids from the sensitive JFH-1 virus were introduced into the resistant J6 virus, no significant change in neutralization sensitivity was observed for any of the variant amino acid groups (Fig. 2). Many previous studies have identified amino acid mutations associated with resistance either alone or in combination with other mutations for monoclonal antibodies (21, 47–49, 54). These studies have shown that mutations both within and outside neutralizing antibody binding epitopes can confer resistance or sensitivity to neutralization by broadly neutralizing monoclonal antibodies. Additionally, a study of a large panel of HCV isolates found that there is extreme variation in neutralization sensitivity to monoclonal antibodies regardless of genotype, and the authors suggest that these differences could be the result of isolate-specific polymorphisms (50). There remains a possibility that amino acid variants not identified in this study or alternative combinations of the identified amino acid variants may also be affecting isolate-specific neutralization between the J6 and JFH-1 virus strains, either alone or in combination with the HVR1. However, we found that HVR1 of the J6 strain conferred resistance to neutralization by our 1a E1/E2 vaccine antisera as well as to various neutralizing monoclonal antibodies, while the JFH-1 HVR1 conferred sensitivity (Fig. 3 and 5). Consistent with previous studies, the removal of the HVR1 increased the neutralization sensitivity of HCV to both broadly neutralizing monoclonal antibodies and to our 1a E1/E2 vaccine antisera (Fig. 3 and 5) (23, 34, 38, 55, 56). The observed differential neutralization of J6 and JFH-1 was not due to our 1a E1/E2 vaccine antisera containing neutralizing antibodies selectively targeting the JFH-1 HVR1 region over that of the J6 HVR1 (Fig. 4). We found that J6 virus infectivity is clearly more sensitive to inhibition by anti-SR-B1 than JFH-1 and that this effect is mediated through HVR1 (Fig. 6). Furthermore, we observed that J6 HVR1 but not JFH-1 HVR1 binds SR-B1 directly (Fig. 7). Our data suggest that SR-B1 interaction with HVR1 is isolate dependent and affects the subsequent neutralization sensitivity of the virus. This agrees with previous research into the interaction of J6 with SR-B1. It has been previously shown using deleted HVR1 proteins that for the J6 virus, HVR1 is critical for the interaction with SR-B1 (31, 34). Direct interaction of deleted HVR1 JFH-1 E2 has not been investigated previously. But JFH-1-soluble E2 was shown to interact with SR-B1 expressed on CHO cells, and cell-culture adaptive mutations that alter CD81 binding change the interaction of JFH-1 with SR-B1 (20, 32, 57). Additionally, for the JFH-1 virus, the SR-B1 has been shown to be interchangeable for entry with the low-density lipoprotein receptor (58). Along with our data, this potentially indicates that the conformation of JFH-1 E2 is important for the interaction with SR-B1, either directly or through interactions mediated by lipoproteins, while this interaction is more strongly mediated by HVR1 for the J6 virus. This interaction of SR-B1 with HVR1 that occurs for the J6 virus could lead to an alteration of the kinetics of entry that allows the J6 virus to enter the cell faster than the JFH-1 virus and thus reduces the exposure time of the virus to neutralizing antibodies, resulting in the observed resistance to neutralization. Previous studies have shown that virus-associated high-density lipoproteins increase HCV infectivity via increased entry kinetics through enhanced interactions with entry receptor, SR-B1, leading to a reduction in antibody neutralization dependent on the presence of HVR1 (23, 59, 60). It is possible that HVR1 of J6 virus could mediate differential interactions with other components (such as lipoproteins) that could affect the kinetics of binding to target cells or the accessibility to neutralizing epitopes directly or a combination of both. Alternatively, our data also agree with recent studies that suggest that HCV envelope proteins could be in a “shielded” or “closed” conformation prior to binding SR-B1 and that interactions with SR-B1 result in a conformational change that then exposes these epitopes, allowing subsequent CD81 binding (61, 62). Our data potentially extend this theory to indicate that HVR1-SR-B1 interactions and induced conformational changes are isolate specific.

The 1a E1/E2 vaccine elicited antibodies that are capable of binding peptides containing the 31 amino acids downstream of the HVR1 of both 2a J6 and JFH-1 (Fig. 4) despite the J6 virus resistance to neutralization. This region of the E2 protein is known to contain conserved residues important for binding of many broadly neutralizing monoclonal antibodies (16). This agrees with our previously published data showing that vaccine-induced antisera compete for binding with broadly neutralizing monoclonal antibodies that target this region as well as several other epitopes throughout E1 and E2 (45). Previous vaccination studies with our 1a E1/E2 vaccine antigen in rodents, goats, chimpanzees, and humans have demonstrated the ability of a 1a E1/E2 vaccine candidate to elicit broadly cross-neutralizing antibodies against most global HCV genotypes and subtypes, although neutralizing antibody responses against genotypes 2 and 3 were lower than those against other HCV genotypes (42, 44–46, 63). Furthermore, recombinant E1/E2 was shown to be safe and immunogenic in human volunteers (43) and remains the only HCV vaccine candidate to have demonstrated efficacy in the chimpanzee model at preventing chronic infection following challenge with either homologous or heterologous 1a virus (40, 41, 64). In contrast, a T cell vaccine comprising an adjuvanted HCV polyprotein lacking E1/E2 failed to demonstrate efficacy at reducing chronic infection despite ameliorating acute viremia and acute hepatitis (64). Therefore, the E1/E2 vaccine represents a promising HCV vaccine candidate when used either alone or in combination with immunodominant T cell antigens to further boost cellular immunity. A combination vaccine comprising E1/E2 from multiple genotypes to overcome resistant HCV strains and the use of stronger adjuvants to boost cross-neutralizing antibody titers targeting conserved viral epitopes is likely to be a valuable strategy based on our current work.

## MATERIALS AND METHODS

### Cell culture.

Huh7.5 cells were grown in Dulbecco’s modified Eagle’s medium (DMEM) (Gibco) supplemented with 10% heat-inactivated fetal bovine serum (FBS) (Sigma-Aldrich), 0.1 mM nonessential amino acids (NEAA) (Invitrogen), 100 units/ml penicillin, and 100 μg/ml streptomycin (Invitrogen) in an incubator supplemented with 5% carbon dioxide (CO_2_) at 37°C.

### Antibodies and antiserum.

The HCV detection antibody mouse anti-NS5A (9E10) was provided by Charlie Rice and Tim Tellinghuisen and has been described previously (65). Envelope protein-specific monoclonal antibodies AR1B, AR2A, AR3A, AR4A, and AR5A were provided by Mansun Law and have been described previously (19, 53). Anti-SR-B1 antibody has been described previously (66). AR1B, AR2A, AR3A, AR4A, and AR5A, anti-SR-B1, and monoclonal mouse anti-CD81 antibody (BD Biosciences) were used to neutralize wild-type (WT) and recombinant cell culture-derived HCV (HCVcc) as described below.

1a E1/E2 vaccine antisera from a goat (G757) immunized with recombinant E1/E2 derived from the genotype 1a HCV-1 strain were described previously (45). Presera collected prior to vaccination and postsera collected after five immunizations adjuvanted with Invivogen’s AddaVax or complete or incomplete Freund’s were used in this study. Complement in the antisera was inactivated at 56°C for 30 min prior to use in neutralization assays.

### HCV plasmids.

Genotype 2a JC1-NS5A-nluc (referred to as J6 in this study) is a chimeric virus composed of the J6 genome (nucleotides 341 to 3430, encoding core to NS2 proteins) and the JFH-1 genome (nucleotides 1 to 340 and 3431 to 9679, 3′ and 5′ untranslated regions and NS3 to NS5B proteins) (65). This virus contains a Nano luciferase (nluc) reporter gene within domain III of the NS5A protein and was provided by Michael Beard and described in reference 67. Genotype 2a JFH-1-NS5A-nluc (referred to as JFH-1 WT in this study) was generated by replacing the fragment between the AvrII and SnaBI restriction sites (nucleotides 3867 to 8450) in the cell culture-adapted JFHrr virus (provided by Rodney Russell and described in reference 68) with the same AvrII and SnaBI fragment from the JC1-NS5A-nluc virus that contains the nluc gene. Fragments were ligated together using T4 DNA ligase (New England Biosciences) following the manufacturer’s protocol.

To generate E1 or E2 hybrid virus constructs, PCR was used to generate fragments of the J6 or JFH-1 E1 and E2 proteins. Fragments were created with overlapping sequences comprising the restriction cut sites of ClaI (at nucleotides 709 and 3931) for the J6-JFH-1 E1 and J6-JFH-1 E2 constructs and FpsI and AvrII cut sites (at nucleotides 11177 and 3867) in the JFH-1-J6 E2 construct. Fragments were ligated together using T4 DNA ligase (New England Biosciences) following the manufacturer’s protocol.

JFH-1 E2 single-amino-acid variants and HVR1-deleted (Δ) constructs in the J6 virus were created using the QuikChange II site-directed mutagenesis kit (Agilent) as described in the manufacturer’s protocol. For constructs that required multiple mutations that could not fit within one primer, constructs were sequentially created for each mutation using the previously constructed mutant as the template for the PCR, and the procedure was repeated until the desired mutations were achieved.

A construct containing all the JFH-1 E2 amino acid variants in the J6 virus was created using infusion cloning (TaKaRa Bio) following the manufacturer’s protocol utilizing a synthetic gBlock gene fragment (Integrated DNA Technologies) for the J6 E2 that contained all the JFH-1 variant amino acids. Infusion cloning (TaKaRa Bio) was also used to generate the HVR1 hybrid viruses with a gBlock gene fragment (Integrated DNA Technologies) created for JFH-1 HVR1 to clone into the J6 virus or J6 HVR1 to clone into the JFH-1 WT virus.

### HCVcc generation.

HCV RNA was electroporated into Huh7.5 cells as previously described (44). Briefly, 5 μg of RNA was electroporated into Huh7.5 cells using the Electro Square Porator ECM 830 (BTX). Cells were allowed to recover at room temperature for 10 minutes and were then plated on p150 dishes. Cells were incubated in a 5% CO_2_ incubator at 37°C. Supernatant virus was collected and filtered with 0.22-μmol filters at 72 and 120 hours postelectroporation. Virus was then aliquoted and stored at –80°C. The 50% tissue culture infectivity dose (TCID_50_)/ml of the virus was calculated as described previously using the anti-NS5A (9E10) antibody to detect foci (65).

### Neutralization and inhibition assays.

The neutralization assay protocol was performed similarly to previously described methods, with modifications noted below (44). Briefly, diluted sera or monoclonal antibody was added to HCVcc diluted to 500 TCID_50_/ml, and the mixture was incubated for 1 hour at 37°C. The virus/antibody mixture was then added to Huh7.5 cells plated on 96-well plates for 6 hours, followed by replacement with fresh growth medium. At 48 hours postinfection, cells were lysed in Nano-Glo luciferase assay buffer and substrate (Promega). The luminescence was measured using the EnSpire 2300 multilabel reader (Perkin-Elmer). The percent neutralization was calculated by subtracting the treatment signal from the presera well signal and dividing by the total possible infection (presera well). Percent neutralization was plotted with GraphPad Prism 7 software. Half-maximal inhibitory concentration (IC_50_) values were determined by finding the nonlinear regression of a variable slope using the software GraphPad Prism 7.

For anti-CD81 and anti-SR-B1 inhibition assays, antibodies were preincubated with Huh7.5 cells for 4 hours. Anti-CD81 was diluted 2-fold (starting with 0.5 μg/ml), and anti-SR-B1 was diluted 5-fold (starting with 10 μg/ml). After 4 hours of preincubation with cells, HCVcc diluted to 500 TCID_50_/ml was added to Huh7.5 cells for 6 hours, followed by replacement with fresh medium. At 48 hours postinfection, cells were lysed, and the luciferase signal was detected as described above. The percent inhibition was calculated by subtracting the treatment signal from the virus-only well signal and dividing by the total possible infection (virus-only well). The percent neutralization was plotted with GraphPad Prism 7 software. Half-maximal inhibitory concentration (IC_50_) values were determined by finding the nonlinear regression of a variable slope using GraphPad Prism 7 software.

For Fc-SR-B1 and Fc protein inhibition assays, proteins (at a concentration of 50 μg/ml) were preincubated with HCVcc diluted to 500 TCID_50_/ml for 1 hour prior to addition to Huh7.5 cells. Cells were infected for 6 hours, followed by replacement with fresh medium. At 48 hours postinfection, cells were lysed, and the luciferase signal was detected as described above.

### Peptide binding enzyme-linked immunosorbent assay.

N-terminal biotinylated peptides of amino acids 384 to 411 (the 27 amino acids of the HVR1) of genotype 1a H77, genotype 2a J6, genotype 2a JFH-1, and amino acids 412 to 443 (the amino acids directly downstream of HVR1) of J6 and JFH-1 as well as the scrambled control of the 2a JFH-1 HVR1 peptide were synthesized by GL Biochem. Peptides were added to neutravidin-coated 96-well plates at 0.5 μg/well for 1 hour. Plates were blocked with 5% bovine serum albumin (BSA). The presera or postvaccination polyclonal E1/E2 antisera from goat 757 were added to wells in 3-fold serial dilutions starting with 1/50. Horseradish peroxidase (HRP)-conjugated anti-goat antibody (Santa Cruz Biotechnology) was used to detect goat antisera binding to peptides. Plates were developed using tetramethylbenzidine (TMB) substrate (Mandel Scientific), and the reaction was stopped after 4.5 minutes. The EnSpire 2300 multilabel reader (Perkin-Elmer) was used to record absorbance values for an optical density (OD) of 450 nm. Nonspecific absorbance values for negative controls were subtracted from experimental wells, and data were plotted using GraphPad Prism 7 software.

### SR-B1 binding enzyme-linked immunosorbent assay.

Plates were coated with the N-terminal biotinylated HVR1 peptides described above in addition to the 3a S52, 4a ED43, 5a SA13, 6a HK6a, and 7a QC69 HVR1 sequence and scramble controls of the 1a H77, 2a J6, and 2a JFH-1 HVR1 peptides at 1 μg/well. J6-soluble E2 protein (provided by Joe Marcotrigiano and described previously [29]) or JFH-1-soluble E2 (purified from an Fc-tagged precursor through affinity chromatography as previously described [69]) was coated directly onto plates at 0.5 μg/well. Plates were blocked in 5% BSA. Recombinant Fc-tagged SR-B1 (Fc-SR-B1) protein (Abcam) was added to wells in a 2-fold serial dilution starting with a 1 μg/ml concentration for HVR1 peptide binding or 10 μg/ml for J6- and JFH-1-soluble E2 binding. An anti-Fc HRP-conjugated antibody (Jackson ImmunoResearch) was used to detect binding of the Fc-SR-B1 protein to wells. Plates were developed and absorbance was read as described above. Data were plotted with GraphPad Prism 7 software. The ability of human Fc protein (Abcam) alone to bind to biotinylated peptides and J6- and JFH-1-soluble E2 was tested following the same protocol described above as a control for the potential Fc tag binding.

### Statistical analysis.

Statistical analysis was by one-way analysis of variance (ANOVA) or two-way ANOVA where appropriate. ANOVA was followed with a Dunnett’s multiple-comparison test to compare significance between groups. All analyses were performed using GraphPad Prism 7 software. *P* values of <0.05 were considered statistically significant.
